# Utility of P63 in Differentiating Giant Cell Tumor from Other Giant Cell-Containing Lesions

**DOI:** 10.5146/tjpath.2021.01538

**Published:** 2022-01-21

**Authors:** Monalisa Hui, Shantveer G Uppin, K Karun Kumar, S Radhika, Patnala Chandrasekhar, K Nageshwara Rao

**Affiliations:** Department of Pathology, Nizam’s Institute of Medical Sciences, Hyderabad, India; Department of Clinical Pharmacology and Therapeutics, Nizam’s Institute of Medical Sciences, Hyderabad, India; Department of Orthopaedics, Nizam’s Institute of Medical Sciences, Hyderabad, India

**Keywords:** Giant cell tumor of bone, Giant cell-containing lesions of bone, p63, Immunohistochemistry

## Abstract

*
Objective:* To assess P63 expression in giant cell-containing lesions of the bone (GCLB) and to determine its utility in differentiating giant cell tumor of the bone (GCTB) from other GCLBs.

*
Material and Method:* Cases diagnosed as GCLB on histopathology were included in the study. P63 immunohistochemistry was performed in all the cases. The percentage of cells showing nuclear positivity was assessed in the non-giant cell component. Statistical analysis was performed using the Mann-Whitney U test.

*
Results:* Of the total 53 cases studied, the majority were GCTBs (23), followed by 12 cases of chondroblastomas (CBL) and 18 other giant cell lesions (GCLs). All giant cell-containing lesions except one case of CBL and brown tumor of hyperparathyroidism (BTH) showed P63 staining in the non-giant cell component. However, the mean P63 labeling of GCT (52.6%) was higher compared to CBL (28.3%), aneurysmal bone cyst (ABC) (15.2%), non-ossifying fibroma (NOF) (24.5%), giant cell lesion of small bones (GCLSB) (11%), BTH (6.8%) and chondromyxoid fibroma (CMF) (12.3%), with a p-value of <0.001.

*
Conclusion:* Although p63 was present in majority of the GCLBs, its percentage positivity was significantly higher in GCTB compared to the other GCLBs. The diagnosis of GCTB is likely if cut-off value of >50% is applied.

## INTRODUCTION

Morphology in correlation with clinical and radiological findings is the cornerstone for the diagnosis of primary bone tumors. The giant cell rich tumors of the bone are morphologically distinct entities which share in common the presence of multinucleated osteoclast-like giant cells (1). With the advent of minimally invasive procedures, the material obtained for initial diagnosis of primary bone tumors is often limited and poses a diagnostic dilemma. Though routine morphology is sufficient in most of the cases, immunohistochemistry (IHC) helps to resolve the diagnostic difficulties that are especially encountered in small biopsies with atypical morphology and ambiguous imaging. Until the advent of anti-histone antibodies, there was no well-established diagnostic marker for giant cell tumor of the bone (GCTB). Studies have shown conflicting results regarding over expression of p63 by IHC and molecular methods in the stromal cells of GCTB (2–5). In this article we have assessed the expression of p63 in giant cell-containing lesions of the bone and determined its utility in differentiating GCTB from other giant cell-containing lesions of the bone (GCLBs).

## MATERIAL and METHOD

The study included non-consecutive histologically verified cases of various giant cell-containing lesions of the bone (GCLB) where paraffin blocks were available for IHC. The clinical features, location and imaging findings were retrieved from the medical records. The diagnosis was made on 42 curettage specimens, 6 open biopsies and 5 resected specimens. The hematoxylin and eosin-stained sections of all the cases were reviewed along with the clinical, imaging and other relevant laboratory findings to confirm the original diagnosis. The appropriate paraffin block was selected for IHC after examining the representative hematoxylin and eosin-stained sections. The decalcified sections, and areas of hemorrhage and necrosis were excluded. IHC was performed on 3-4µm thick sections using mouse monoclonal antibody against p63 (Pre-diluted, Ready to Use Antibody, Biogenex).The percentage of nuclear positivity was assessed in non-giant cell component after counting a minimum of 500 nuclei in the hot spots. The intensity of staining was evaluated as weak (+1), moderate (+2) and strong (+3). Moderate to strong intensity nuclear staining in >1% of the cells was considered positive. Scoring was applied by two pathologists independently and the average of the two scores was taken into account. IHC was performed in batches and slides with a positive control were included in every batch. Statistical analysis was performed using the Mann-Whitney U test, and a p-value of <0.05 was considered significant. A receiver operating characteristic (ROC) curve analysis was done to determine the cut-off value of p-63 positivity in order to predict the diagnosis of GCTB. Both the tests were done using SPSS software version 20.

## RESULTS

Of the total number of 53 cases studied, the majority were GCTBs (23), followed by 12 cases of chondroblastoma (CBL). The other GCLBs studied included 6 aneurysmal bone cysts (ABC), 3 cases of non-ossifying fibroma (NOF), in addition to 2 cases each from brown tumor of hyperparathyroidism (BTH), giant cell lesion of small bones (GCLSB) and chondromyxoid fibroma (CMF) and a 1 case each of giant cell rich reparative granuloma (GCRG), osteoblastoma and telangiectatic osteosarcoma.

**Figure 1:**
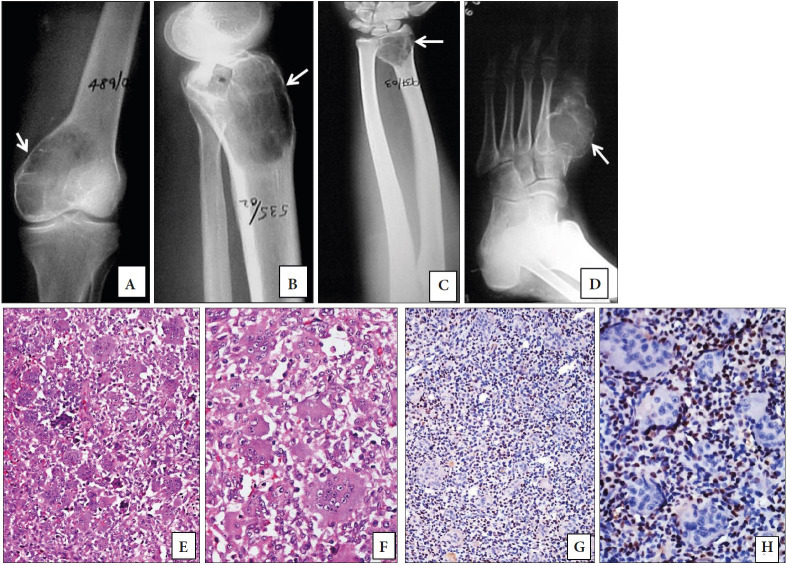
Plain radiographs of giant cell tumor of bone (GCTB) presenting as expansile lytic lesions (white arrows) involving the epiphysis of **A)** distal femur, **B)** proximal tibia, **C)** distal radius and **D)** first metatarsal. **E, F)** Histological sections of GCTB showing spatial distribution of osteoclast-like giant cells and mononuclear cells (H&E; E; x200, F; x400). **G, H)** Diffuse strong nuclear p63 staining in the mononuclear cells with sparing the nuclei of osteoclast-like giant cells (p63 antibody; G; x200, H; x400).

Regarding the 23 GCTBs, the age of the patients ranged from 14 to 69 years with a mean age of 30.18 years. There was a slight male predominance with a M:F ratio of 1.3:1. The presentation was with pain and swelling in the distal femur and proximal tibia in 18 patients, the distal radius in 2 patients and one case each of the base of proximal phalanx of the right ring finger, the left third metacarpal and the proximal humerus. The plain radiographs of GCTB involving various sites are illustrated in Figure 1(A-D). The duration of the symptoms ranged from one month to 18 months. On histopathology, all showed a characteristic biphasic pattern with spatial arrangement of the osteoclast giant cells amidst the mononuclear cells as shown in Figure 1(E and F). The nuclei of the mononuclear cells resembled the giant cells, which were large and had 40 to 50 nuclei. There was no clustering of giant cells. Osteoid formation was not seen. Aneurysmal bone cyst-like changes were noted in 7 cases. However, benign fibrous histiocytoma-like areas were not seen in any of the cases.

**Figure 2:**
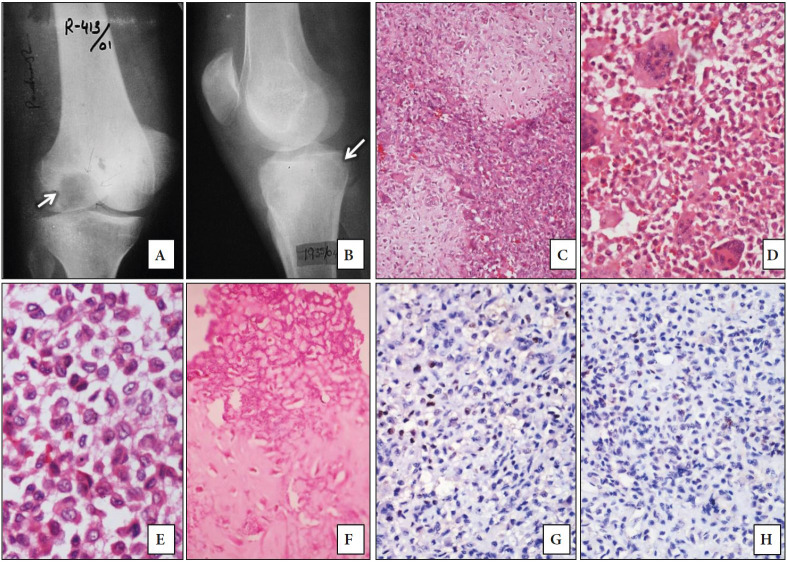
**A, B)** Plain radiographs of Chondroblastomas (CBL) showing a well-defined lytic lesions involving epiphysis of distal femur and proximal tibia. **C-F)** Histological sections of CBL showing lobules of eosinophilic cartilaginous matrix with intervening cellular areas. These cellular areas show sheets of polygonal shaped chondroblasts and osteoclast-like giant cells. On higher magnification the chondroblasts have round oval vesicular which show indentations and longitudinal grooves. Areas of pericellular chicken-wire calcification can be noted in (F). (H&E; C; x100, D; x400, E; x1000, F; x400). **G, H)** Moderate intensity nuclear staining for p63 in some of the mononuclear cells (p63 antibody; x400).

Regarding the 12 CBLs, the age of the patients ranged from 12 to 35 years with a mean age of 18.1 years and M:F of 1.4:1. The majority were located in the distal femur (4 cases) followed by the proximal tibia (3 cases) and the proximal femur (2 cases). There was one case each located in the distal fibula, calcaneum and manubrium sterni. The duration of the symptoms ranged from 2.5 months to 3 years. On histopathology, the osteoclast-like giant cells were randomly distributed. The mononuclear cells were uniform round to polygonal with well-defined cytoplasmic borders and longitudinal nuclear grooves. Pink hyaline cartilage and pericellular lace-like chicken wire calcifications were also noted. Aneurysmal bone cyst-like changes was noted in 2 cases. The plain radiographs and histopathological findings of CBL are illustrated in Figure 2(A-F).

**Figure 3:**
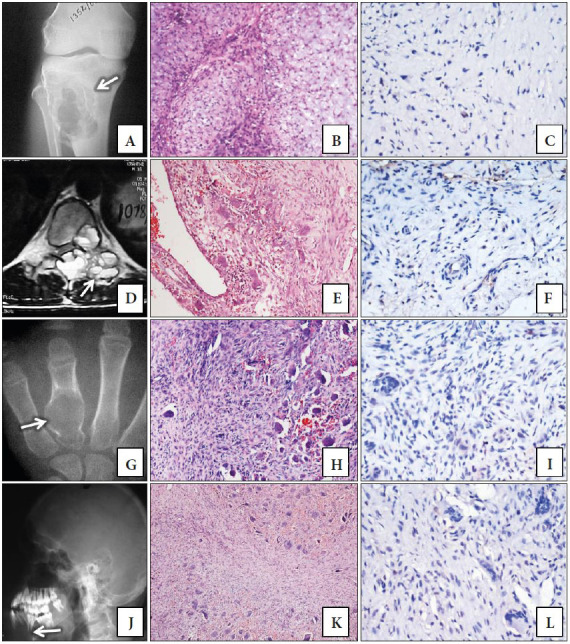
**A)** Plain radiograph of chondromyxoid fibroma (CMF) showing well defined expansile eccentric lytic lesion involving proximal metaphysis of tibia. **B)** Histological section of CMF showing lobules with central hypocellular and peripheral cellular areas with chondromyxoid matrix in the background. Central areas contain bland spindle to stellate cells in a chondromyxoid background. The peripheral areas show plump spindle to polygonal cells along with few osteoclast-like giant cells. (H&E; x100). **C)** Weak p63 staining is seen in scattered cells. **D)** MRI spine of aneurysmal bone cyst reveals an expansile osteolytic lesion involving posterior elements of vertebra with classic internal AIR-fluid levels. **E)** Histological section of ABC showing cystic space separated by septa containing bland spindle cells and osteoclast-like giant cells (H&E; x100). **F)** Weak nuclear staining for p63 in scattered spindle cells. (p63 antibody; x 400).** G)** Plain radiograph of giant cell lesion of small bone (now considered to be solid ABC) presenting as well defined lytic lesion in the metacarpal bone. **H)** Histological sections showing non-uniformly distributed giant cells amongst bland spindle shaped cells along with focal haemorrhage (H&E; x100). **I)** Weak staining for p63 noted in few cells while rest of the cells are negative (p63 antibody; x400). **J)** Plain radiograph of brown tumor of hyperparathyroidism (BTH) presenting as lytic lesion in the centre of mandible; **K)** Histological section showing non-uniformly distributed giant cells with haemorrhage and fibrosis (H&E; x100). **L)** Most cells are negative for p63, with only weak staining in scattered cells. (p63 antibody; x400).

The mean age of the ABC patients was 21 years and the lesions were primarily located in the humerus (3 cases), the vertebral bodies (2 cases) and the proximal femur (1 case). On microscopy, there were blood filled cystic spaces separated by fibrous septae containing osteoclast-like giant cells and proliferation of fibroblasts along with reactive woven bone rimmed by osteoblasts. The three NOF patients presented with a lytic lesion in the tibia and femur. The two cases of giant cell lesion of the small bones (fourth metacarpal and middle phalanx of the right middle finger) are now considered as solid ABC whereas the term GCRG of jaw (1 Case) is still retained as it is in the recent World Health Organization classification of soft tissue and bone (6). The giant cells showed clustering with fewer nuclei as opposed to the uniform distribution of the giant cells in GCTB. Both the cases of CMF were located in the left tibia. The two cases of BTH were located in the mandible and left tibia. These patients had elevated serum calcium and parathormone levels and were later found to have parathyroid adenomas. The imaging and histopathological findings of various giant cell-containing lesions are shown in Figure 3(A, B, D, E, G, H, J and K). A single case of osteoblastoma was located in the L4 vertebral body and a case of telangiectatic variant of osteosarcoma involved the left occipital bone.

All the GCTBs showed strong nuclear positivity in the stromal cells and are depicted in Figure 1(G and H). The percentage positivity of cells displaying p63 immunostaining ranged from 50.5% to 71 % except for one case located in the distal femur that had a positivity of 14%. None of the cases showed any evidence of nuclear staining in the multinucleate giant cells. All the other GCLBs except one case each of CBL and BTH showed p63 staining in the non-giant cell component/ stromal cells. Out of the 11 cases of CBL that were positive for p63, 9 cases had weak to moderate intensity staining in less than 50% of the cells as shown in Figure 2(G and H). The mean p63 labeling in GCTB (56.2%) was much higher compared to CBL (28.3%), ABC (15.2%), NOF (24.5%), GCLSB (11%), BTH (6.8%) and CMF (12.3%). A single case each of osteoblastoma, GCRG and telangiectatic osteosarcoma showed nuclear staining in 52.5%, 45% and 34.5% of the cells respectively. The p63 positivity was found to be statistically significant in patients with GCTB when compared to non-GCTB as analyzed by the Mann-Whitney U test (U=46.5, p<0.001). ROC analysis showed a cut off value of 49.75 for p63 and had a sensitivity of 95% and specificity of 90% to diagnose GCTBs with an area under curve (AUC) of 93.3%, p <0.001. The staining of p63 in CMF, ABC, GCLSB and BTH are shown in Figure 3C, 3F, 3I and 3L respectively. The location and distribution of p63 positive staining cells in GCTB and various GCLBs are provided in Table 1.

**Table 1:** Distribution and p-63 staining of all the giant cell-containing lesions.

**Giant cell-containing lesions of the bone**	**Number of cases**	**Location**	**p63 IHC - mean and SD (%)**
Giant cell tumor	23	Femur-10 Tibia-7 Radius-2 Fibula-1 Humerus-1 Fingers-2	56.2±10.7
Chondroblastoma	12	Distal femur-4 Proximal tibia-3 Proximal femur-2 Distal femur-1 Calcaneum-1 Sternum-1	28.3±19.5
Aneurysmal bone cyst	6	Humerus-3 Vertebrae-2 Femur-1	15.2±3.8
Non-ossifying fibroma	3	Tibia-2 Femur-1	24.5 ±11.1
Giant cell reparative granuloma	1	Jaw	45
Giant cell lesion of the small bones (solid variant of aneurysmal bone cyst)	2	Fourth metacarpal-1 middle phalanx of right middle finger-1	11±5.4
Chondromyxoid fibroma	2	Tibia-2	12.3 ±3.9
Brown tumor of hyperparathyroidism	2	Mandible-1 Tibia-1	6.8 ±8.8
Osteoblastoma	1	L4 vertebrae	52.5
Telangiectatic Osteosarcoma	1	Left occipital bone	34.5

## DISCUSSION

GCLBs are a heterogeneous group of tumors and tumor-like lesions of the bone with a wide range of differential diagnosis. Definite diagnosis is challenging in the setting of limited sampling, unusual age and location at presentation. The morphology of the mononuclear cells gives a clue to the diagnosis. However, the key diagnostic component may be under represented in biopsy. Secondary changes like ABC which frequently accompanies GCTB or CBL may obscure original morphology and overshadow the underlying primary tumor in biopsy specimens (7,8).

This study showed p63 expression in all cases (23/23) of GCTB. Almost all the cases except one showed more than 50% nuclear positivity. The intensity of the staining was strong and was limited to the mononuclear cells. Similar to our study, Hammas, Dickson and Linden also reported overexpression of p63 in all GCTB (2,9,10). De La Rosa G, Paula and Lee reported p63 overexpression in 86.9%, 82% and 81% of the cases respectively (3–5). Yanagisawa reported higher mean p63-positivity for recurrent GCTB (73.6%) compared to non-recurrent cases (29.1%) (11). However, its usefulness as a prognostic marker in recurrence has not been evaluated in our study.

Studies have shown variable expression of p63 in CBL ranging from 30% to 83.3% (2–5). Dickson found expression of p63 in 30% of the cases with a mild to moderate staining intensity in 7-75 % of the cells (2). Although De la Roza observed p63 expression in 10 out of 12 cases, the intensity of staining was weak to moderate except in one case (4). In contrast to strong nuclear staining observed in GCTB, a weak to moderate intensity staining involving less than 50% cells were seen in 9 out of the 11 cases of CBL that showed p63 positivity. The rate of p63 positivity in ABC was much higher compared to the findings of Hammas (40%), Lee (20%), Dickson (28.6%), Paula (51%) and De la Roza (62.5%) (2–5,10).

Although GCTB affects a relatively older population, there is often considerable overlap between the clinical features of GCTB and CBL. GCT has also been documented in children and adolescents with biological behavior similar to that seen in adults, except a marked female predominance. The presence of an open physis does not impede the tumor to involve the epiphyseal cartilage (12). On the other hand, CBL in adults more frequently involves the flat bones and short bones of the hands/feet with an aggressive behavior compared to children (13). As both the tumors are located in the epiphyseal region, absence of a chondroid matrix often causes confusion. To differentiate the above entities, Lee recommends the use of S100 along with p63. A strong nuclear p63 staining with weak S100 in the mononuclear cells favors GCTB over CBL (3). Akpalo reported DOG1 as a highly sensitive and specific marker for CBL (14).

The other giant cell-containing lesions like ABC, NOF, GCLSB, and BTH showed positivity for p63 in all cases but percentage of positivity and intensity of staining was significantly lower than that of GCTB involving less than 50% of the cells. Expression of p63 in most of the GCLBs may lower its specificity as a diagnostic marker. Hence a 50% cut-off value can be used to improve the specificity that would reliably distinguish GCTB from other GCLBs after taking into consideration the age and location of the tumors. A similar suggestion was also made in the Paula study (5).

The morphology of GCRG closely resembles BTH. A careful clinical history of hyperparathyroidism helps in differentiating these two entities. All the other studies except De la Roza have shown negative immunostaining for p63 in all the cases of central giant cell granuloma (CGCG) reflecting a pathogenesis different from GCTB (2–4,10,15). The latter has shown p63 positivity in all the four cases of CGCG (4). The single case of CGCG in our study was also positive for p63 but the proportion of cells stained were less than 50%. The number of cases of GCRG, osteoblastoma and telangiectatic osteosarcoma included were very low and this is a limitation of this study.

There is disagreement amongst the various authors regarding the utility of p63 as a diagnostic marker in GCTB. De la Roza showed no difference in p63 positivity by immunostaining among the giant-cell-rich lesions such as GCTB and CBL (4). Our results were consistent with the reports of Hammas, Lee, Paulo et al and Dickson and we suggest its use as a diagnostic marker provided with a cut off value of 50% (2,3,5,10). However Dickson and Lee considered 5% and 10% of cells respectively for cut off (2,3). On the other hand, de La Roza considered any nuclear staining of p63 as positive (4). The discrepancies in staining may be attributed to the antibody clones and antigen retrieval methods. Gene expression profiling have also substantiated the above findings with over expression of p63 in the majority of GCTBs and only a minor fraction of other GCLBs (2,3).

Recent studies have identified H3 histone family member 3A (H3F3A) (G34W/V/R/L) mutations in the majority of GCTBs and H3 histone family member 3B (H3F3B) (K36M) mutations in nearly all CBLs, but these are absent in other GCLBs. IHC using mutation-specific H3G34W and H3K36M antibodies is highly specific for GCTB and CBL respectively and can be used as a diagnostic tool in limited biopsies (16–19). The presence of alternate H3F3A mutations on Sanger sequencing further enhances the diagnostic yield in a subset of GCTB which are negative for H3G34W on IHC (20). The majority of primary ABCs harbor clonal rearrangements of the USP6 gene locus. Cases without the USP6 gene rearrangement hint at the presence of morphologically undetected components of GCTB and CBL in small biopsies (21). However, these novel diagnostic techniques require expertise, standardization and validation which are not feasible in the setting of limited resources and are presently not widely available.

It is also important to differentiate GCTB from other GCLBs as Denosumab has specific therapeutic implications for GCTB and radiofrequency ablation for CBL. These can be used as treatment options alternative to surgical resections (22,23).

## CONCLUSION

Though P63 expression can be seen to a variable extent in all GCLBs of the bone, the percentage of positivity in GCTB is significantly high compared to other GCLBs. Hence p63 staining by IHC with a cut-off of 50% can be used as an additional marker to differentiate GCTB from other GCLBs of bone.

## Conflict of Interest

The authors have no conflict of interest.

## References

[ref-1] Rosenberg A. E., Nielsen G. P. (2001). Giant cell containing lesions of bone and their differential diagnosis. Current Diagnostic Pathology.

[ref-2] Dickson Brendan C., Li Shu-Qiu, Wunder Jay S., Ferguson Peter C., Eslami Behnam, Werier Joel A., Turcotte Robert E., Kandel Rita A. (2008). Giant cell tumor of bone express p63. Mod Pathol.

[ref-3] Lee Cheng-Han, Espinosa Inigo, Jensen Kristin C., Subramanian Subbaya, Zhu Shirley X., Varma Sushama, Montgomery Kelli D., Nielsen Torsten O., Rijn Matt Van de, West Robert B. (2008). Gene expression profiling identifies p63 as a diagnostic marker for giant cell tumor of the bone. Mod Pathol.

[ref-4] Roza Gustavo De la (2011). p63 expression in giant cell-containing lesions of bone and soft tissue. Arch Pathol Lab Med.

[ref-5] Maues De Paula André, Vasiljevic Alexandre, Giorgi Roch, Gomez-Brouchet Anne, Aubert Sébastien, Leroy Xavier, Duval Hélène, Pinieux Gonzague De, Bouvier Corinne (2014). A diagnosis of giant cell-rich tumour of bone is supported by p63 immunohistochemistry, when more than 50 % of cells is stained. Virchows Arch.

[ref-6] Flanagan AM, Laousserie F, O’Donnell PG, Yoshida A, Board WHO Classification of Tumours Editorial (2022). Giant cell tumour of bone. Soft Tissue and Bone Tumours.

[ref-7] Rehkämper Jan, Steinestel Konrad, Jeiler Birte, Elges Sandra, Hekeler Elena, Huss Sebastian, Sperveslage Jan, Hardes Jendrik, Streitbürger Arne, Gosheger Georg, Wardelmann Eva, Baumhoer Daniel, Trautmann Marcel, Hartmann Wolfgang (2018). Diagnostic tools in the differential diagnosis of giant cell-rich lesions of bone at biopsy. Oncotarget.

[ref-8] Zambo Iva, Pazourek Lukáš (2017). [Giant cell-rich lesions of bone and their differential diagnosis]. Cesk Patol.

[ref-9] Linden MD (2009). Giant cell lesions of bone and soft tissues: Diagnostic value of immunohistochemistry (abstract). Mod Pathol.

[ref-10] Hammas Nawal, Laila Chbani, Youssef Alaoui Lamrani My, Hind El Fatemi, Harmouch Taoufiq, Siham Tizniti, Afaf Amarti (2012). Can p63 serve as a biomarker for giant cell tumor of bone? A Moroccan experience. Diagn Pathol.

[ref-11] Yanagisawa Michiro, Kakizaki Hiroshi, Okada Kyoji, Torigoe Tomoaki, Kusumi Tomomi (2013). p63 as a prognostic marker for giant cell tumor of bone. Ups J Med Sci.

[ref-12] Puri Ajay, Agarwal Manish G., Shah Mandip, Jambhekar Nirmala A., Anchan Chetan, Behle Sanica (2007). Giant cell tumor of bone in children and adolescents. J Pediatr Orthop.

[ref-13] John Ivy, Inwards Carrie Y., Wenger Doris E., Williams Don D., Fritchie Karen J. (2020). Chondroblastomas presenting in adulthood: a study of 39 patients with emphasis on histological features and skeletal distribution. Histopathology.

[ref-14] Akpalo Hana, Lange Claudia, Zustin Jozef (2012). Discovered on gastrointestinal stromal tumour 1 (DOG1): a useful immunohistochemical marker for diagnosing chondroblastoma. Histopathology.

[ref-15] Hosur Mahadevi B., Puranik Rudrayya S., Vanaki Shreenivas S., Puranik Surekha R., Ingaleshwar Pramod S. (2018). Clinicopathological profile of central giant cell granulomas: An institutional experience and study of immunohistochemistry expression of p63 in central giant cell granuloma. J Oral Maxillofac Pathol.

[ref-16] Cleven Arjen H. G., Höcker Saskia, Briaire-de Bruijn Inge, Szuhai Karoly, Cleton-Jansen Anne-Marie, Bovée Judith V. M. G. (2015). Mutation Analysis of H3F3A and H3F3B as a Diagnostic Tool for Giant Cell Tumor of Bone and Chondroblastoma. Am J Surg Pathol.

[ref-17] Kervarrec Thibault, Collin Christine, Larousserie Frédérique, Bouvier Corinne, Aubert Sébastien, Gomez-Brouchet Anne, Marie Béatrice, Miquelestorena-Standley Elodie, Le Nail Louis Romée, Avril Pierre, Christophe Pagès Jean, Pinieux Gonzague De (2017). H3F3 mutation status of giant cell tumors of the bone, chondroblastomas and their mimics: a combined high resolution melting and pyrosequencing approach. Mod Pathol.

[ref-18] Girolami Ilaria, Mancini Irene, Simoni Antonella, Baldi Giacomo Giulio, Simi Lisa, Campanacci Domenico, Beltrami Giovanni, Scoccianti Guido, D’Arienzo Antonio, Capanna Rodolfo, Franchi Alessandro (2016). Denosumab treated giant cell tumour of bone: a morphological, immunohistochemical and molecular analysis of a series. J Clin Pathol.

[ref-19] Ogura Koichi, Hosoda Fumie, Nakamura Hiromi, Hama Natsuko, Totoki Yasushi, Yoshida Akihiko, Ohashi Shoko, Rokutan Hirofumi, Takai Erina, Yachida Shinichi, Kawai Akira, Tanaka Sakae, Shibata Tatsuhiro (2017). Highly recurrent H3F3A mutations with additional epigenetic regulator alterations in giant cell tumor of bone. Genes Chromosomes Cancer.

[ref-20] Schaefer Inga-Marie, Fletcher Jonathan A., Nielsen G. Petur, Shih Angela R., Ferrone Marco L., Hornick Jason L., Qian Xiaohua (2018). Immunohistochemistry for histone H3G34W and H3K36M is highly specific for giant cell tumor of bone and chondroblastoma, respectively, in FNA and core needle biopsy. Cancer Cytopathol.

[ref-21] Oliveira Andre M., Hsi Bae-Li, Weremowicz Stanislawa, Rosenberg Andrew E., Dal Cin Paola, Joseph Nora, Bridge Julia A., Perez-Atayde Antonio R., Fletcher Jonathan A. (2004). USP6 (Tre2) fusion oncogenes in aneurysmal bone cyst. Cancer Res.

[ref-22] Rybak Leon D., Rosenthal Daniel I., Wittig James C. (2009). Chondroblastoma: radiofrequency ablation--alternative to surgical resection in selected cases. Radiology.

[ref-23] Puri A., Gulia A., Hegde P., Verma V., Rekhi B. (2019). Neoadjuvant denosumab: its role and results in operable cases of giant cell tumour of bone. Bone Joint J.

